# A Simple and Versatile 2-Dimensional Platform to Study Plant Germination and Growth under Controlled Humidity

**DOI:** 10.1371/journal.pone.0096730

**Published:** 2014-05-07

**Authors:** Tom Sizmur, Kara R. Lind, Saida Benomar, Hannah VanEvery, Ludovico Cademartiri

**Affiliations:** 1 Department of Materials Science & Engineering, Iowa State University, Ames, Iowa, United States of America; 2 Ames Laboratory, US Department of Energy, Iowa State University, Ames, Iowa, United States of America; 3 Department of Chemical & Biological Engineering, Iowa State University, Ames, Iowa, United States of America; University of Nottingham, United Kingdom

## Abstract

We describe a simple, inexpensive, but remarkably versatile and controlled growth environment for the observation of plant germination and seedling root growth on a flat, horizontal surface over periods of weeks. The setup provides to each plant a controlled humidity (between 56% and 91% RH), and contact with both nutrients and atmosphere. The flat and horizontal geometry of the surface supporting the roots eliminates the gravitropic bias on their development and facilitates the imaging of the entire root system. Experiments can be setup under sterile conditions and then transferred to a non-sterile environment. The system can be assembled in 1-2 minutes, costs approximately 8.78$ per plant, is almost entirely reusable (0.43$ per experiment in disposables), and is easily scalable to a variety of plants. We demonstrate the performance of the system by germinating, growing, and imaging Wheat (*Triticum aestivum*), Corn (*Zea mays*), and Wisconsin Fast Plants (*Brassica rapa*). Germination rates were close to those expected for optimal conditions.

## Introduction

Approximately 97% of the calories consumed by humans originate from plants [1]. Recent estimates indicate that the food supply will have to increase by approximately 70% by 2050 to match demand [1]. However, even optimistic estimates predict only a 50% increase in crop yield by 2050 [2].

Improving our understanding of seed germination and root growth could be necessary to ensure our food security in the future, since the germination, emergence, and early establishment of seedlings have a large effect on agricultural yields, especially if below a critical level [3]. Low germination rates reduce crop density, which results in indirect yield loss. Late emergence can result in poor plant performance and a direct yield loss [4], because roots are inadequately established and have less access to water and nutrients during later stages of vegetative and reproductive growth.

Tests of seed viability and vigor typically employ paper to act as a support and to supply moisture: seeds are placed over moist germination paper (and often covered with a second sheet) and incubated. A germination table (also known as a Copenhagen table or Jakobson apparatus) can germinate several seeds simultaneously under one set of conditions [5], [6]: filter paper wicks moisture from a temperature-controlled water tank and provides a flat, horizontal surface on which germination can be observed. However, germination tables are expensive, not universally available, and do not provide control of conditions to individual replicates. Furthermore, they are not ideally compatible with – and never used for – the study of plant root growth. Most plants grown for research purposes are transplanted at least once after germination.

Roots are responsible for the vast majority of the water and nutrient supply to the plant [7], they establish synergic interactions with soil biota [8], [9], and they anchor the plant to the soil [10]. By these functions, the roots influence the growth of the plant and its resilience against environmental stresses such as drought. Root architecture (i.e. its size and structure) plays a fundamental role in plant productivity and crop yield [11]. Nonetheless, roots and their development are one of the most complex and relatively unexplored aspects of the food supply problem [12].

Seedlings are grown in granular media (e.g., soil, sand, perlite, vermiculite) or homogeneous media, such as water (hydroponics), air (aeroponics), or gels (e.g., agar, gelatine, gellan gum). Gels provide a 3D growth environment for the roots, but they otherwise poorly represent the mechanical and structural properties of soils [13], and may expose plants to anoxic conditions [14]. Analysis of the size and structure of a 3D root system requires relatively sophisticated equipment and cumbersome image analysis [15]. Granular media (e.g., soil, sand, vermiculite) is structurally closer to soil, but is opaque to most forms of radiation. The imaging of root systems in those environments requires expensive equipment (X-ray computed tomography or magnetic resonance imaging [16], [17]) that is not widely available, currently has low throughput (individual scans can take hours), and cannot routinely or accurately distinguish live roots from dead organic matter [18]. Roots can be imaged growing against a transparent surface in soil-filled 2D root-boxes called rhizotrons [19]. However, even when tilted at a 43° angle to encourage the roots to grow against the transparent surface, less than half of the total root length is visible [20], root density is overestimated [21], root development is affected by gravitropism, and the soil/glass interface is unlikely to be representative of real soil structure.

The study of both germination and root development in the same environment is experimentally and logistically difficult, because of the lack of convenient and yet highly controlled and capable environments in which to study these processes [22]. Conditions for experimental plant germination and growth must be precise and uniform, because plants are highly sensitive to environmental conditions, and exhibit phenotypic plasticity to a vast array of abiotic stimuli [23]. Some of these conditions (e.g. temperature, light, humidity and CO_2_ concentration) can be controlled using growth chambers [24]. Growth chambers are not ideal environments to study the effect of humidity on plant development because (i) they cannot control humidity of individual replicates, (ii) they expose the plant to the atmosphere and potential contamination, and (iii) they are expensive and not universally available. Therefore, laboratory studies of plant germination and growth under controlled humidity conditions typically require a large upfront investment. These barriers are bound to inhibit or prohibit investigators from other disciplines or developing nations from entering into this area of science.

We describe in this paper an experimental setup for the study of germination and root development of a variety of plants (as shown here, *Brassica rapa*; Wisconsin Fast Plants; Astroplants, *dwf1* [25], *Triticum aestivum*; Wheat, and *Zea mays*; Corn). The platform displays the following capabilities and characteristics: (i) It constantly exposes the plant is to a nutrient solution and to a controlled humidity (ranging between ∼56% and ∼91% in each setup). (ii) It can be used on any laboratory bench, as long as uniform illumination and temperature are provided. (iii) It is composed of reusable or inexpensive parts. (iv) It is scalable to virtually any plant size. (v) It allows imaging of the shoot and root. (vi) It eliminates the gravitational bias on root development by growing the roots on an horizontal and flat 2D surface, which facilitates the imaging and analysis of the entire root system architecture.

## System Design

**Figure 1:**
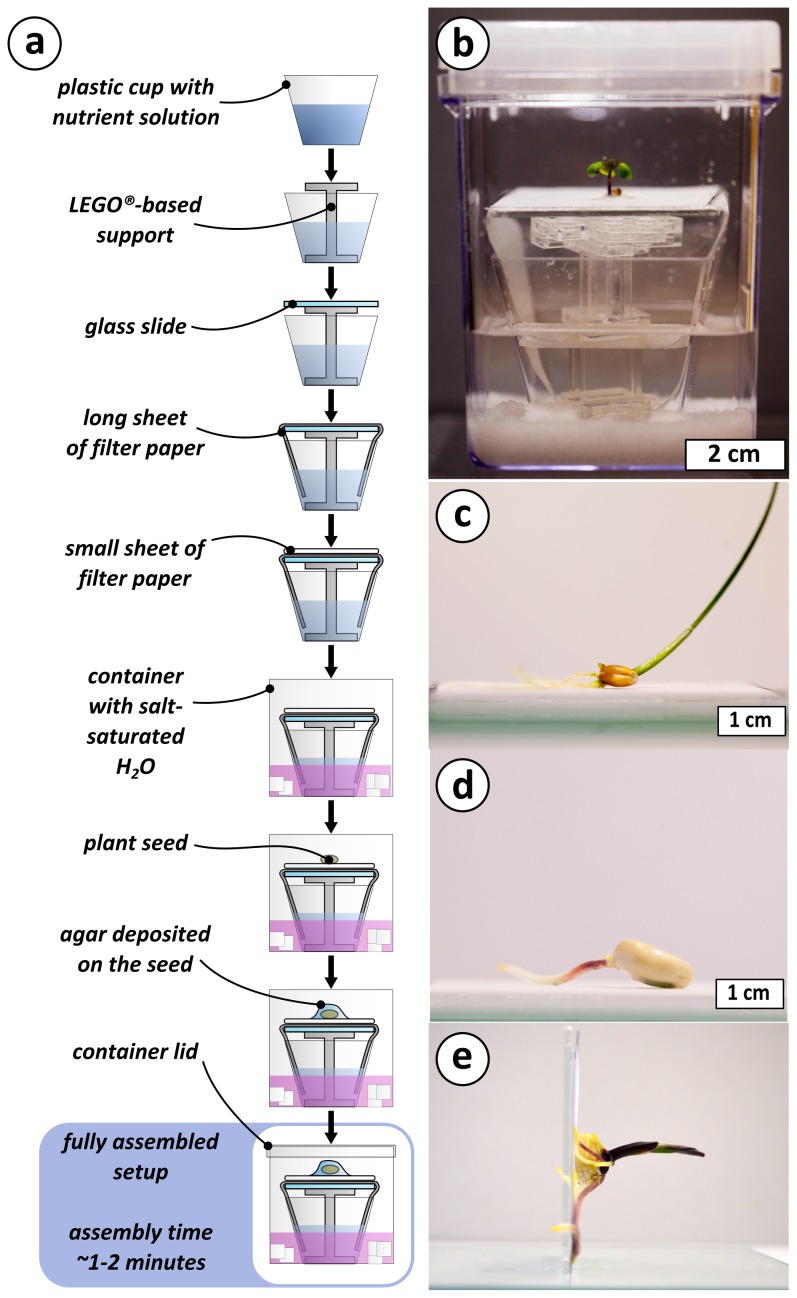
Schematics and picture of plant germination platform. **a**) Scheme of the assembly of the plant germination and growth platform. Pictures of the assembled setup growing (**b**) *Brassica rapa*; Wisconsin Fast Plants; Astroplants, dwf1, (**c**) *Triticum aestivum* (Wheat), and (**d**) *Zea mays* (Corn). **e**) Picture of a Corn seedling held at 90 degrees on paper, demonstrating the anchoring of the roots to the filter paper support.

The assembly of the platform is shown in Figure 1a. It consists of 8 steps that can be completed in approximately 1 to 2 minutes (see Supporting Information S1 for a detailed description and Movie S1 and Movie S2 for a video demonstration) and result in the self-contained plant growth environment shown in Figure 1b.

**Figure 2:**
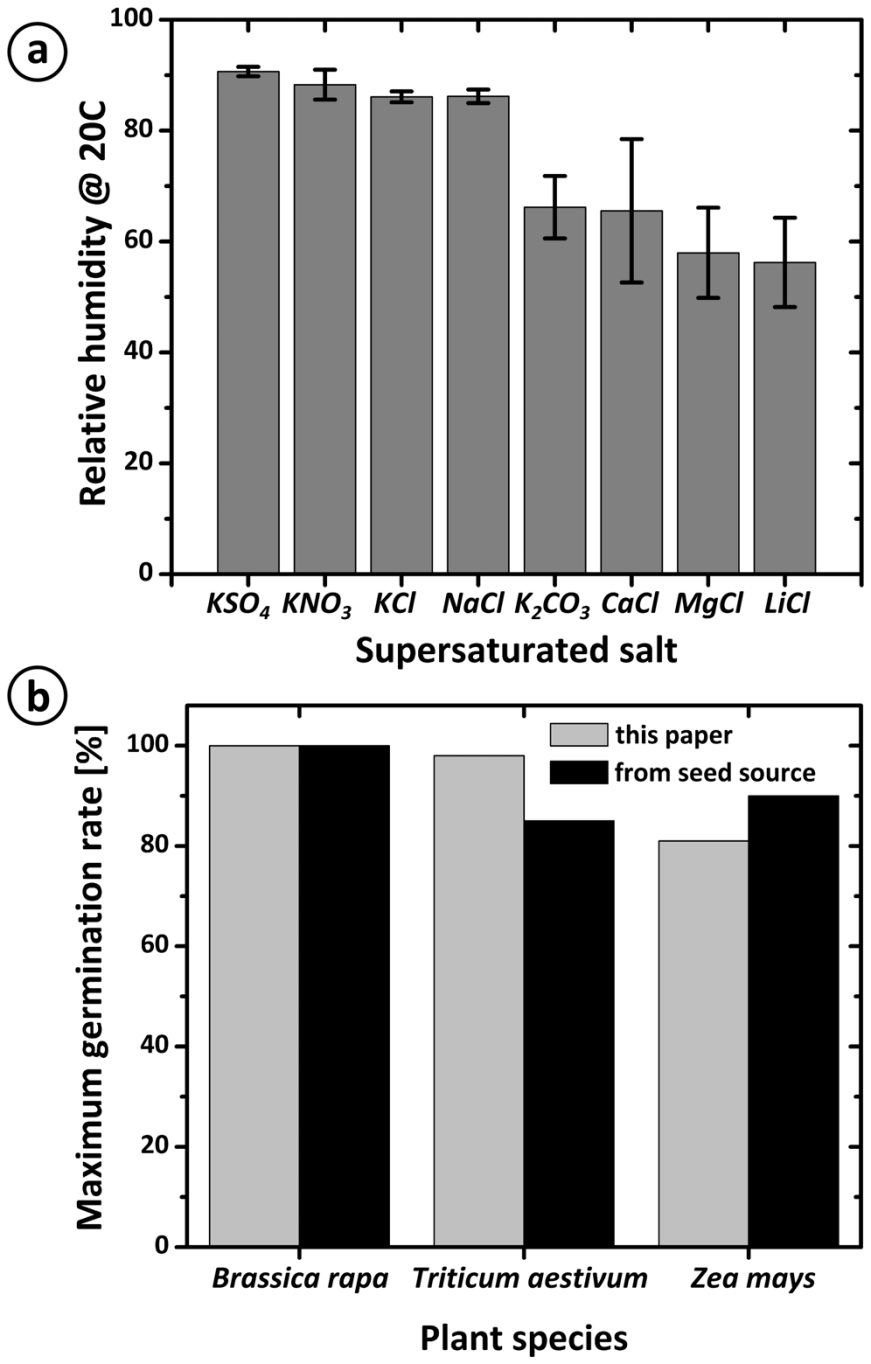
Performance of the germination and growth platform. **a**) Relative humidity in the setup as a function of the salt used to form the supersaturated solution in the reservoir. Error bars are 3 standard errors, n = 3. **b**) Plot of the maximum germination rates obtained for Fast Plants, Wheat and Corn in our platform, compared to optimal germination rates reported by our seed source.

The design of the platform was constrained by a stringent set of conditions. ***Delivery of nutrients and moisture to the seed/plant*** *.* In our setup, the seed (*B. rapa*, *T. aestivum*, or *Z. mays*) is supported on a flat sheet (the “growth sheet”) of filter paper (Whatman #1). The growth sheet lies on top of a larger sheet (the “pump sheet”) of filter paper (Whatman #1) that wicks nutrient solution from an underlying reservoir. The pump sheet imbibes the growth sheet with the nutrient solution. Coating the newly sown seed with a hydrogel droplet (50 µl of gellan gum) improves germination rates: the hydrogel draws water from the filter paper and ensures the seed is moist without eliminating the access to oxygen. ***Compatibility with both germination and growth*** **.** The setup is easily scalable. Figure 1b-d show that three plants with different seed size can be germinated in our platform. The overall scale of the experiment can be controlled to match the size of the plant after the intended growth period (see Supporting Information S1). Plant roots anchor to the filter paper. As shown in Figure 1e, plants grown for 2-3 weeks can be turned sideways without toppling over. ***Control of humidity***. Supersaturated salt solutions in a closed environment establish an atmosphere of known relative humidity [26]. Different salts controlled the relative humidity of the air 5 cm above the growth sheet between ∼56% and ∼91% at 25°C (Figure 2a). The external container (containing the salt solution) is never in contact with the nutrient solution, so neither the salt nor the container can contaminate the paper on which the plant is grown. ***Exclude the influence of gravity on the direction of plant root growth*** *.* Gravity affects root growth by creating a gradient of auxin across the root tip. Auxin is a hormone that inhibits the expansion of root cells. A gradient of auxin across the root cross-section cause the root to bend due to differential expansion of the tissue [27], [28]. The gradient of auxin is determined by the angle between the root tip and the gravitational field: if the root is pointing downward the angle is zero, there is no cross-sectional gradient of auxin, and the root grows mostly straight. Therefore, gravitropism cannot influence the direction of root growth in a horizontal plane. Gravitropism typically dominates the early stages of root growth and can complicate the assessment of the influence of other stimuli (e.g. water or nutrient gradients) on the development of roots: as the root grows, the angle it makes with the gravitational field can change, therefore changing the distribution of auxin in time and space. Roots grown on a horizontal surface still develop a gradient of auxin (the gravitational field is still present), but it remains constant and homogeneous across the whole root system. Therefore, a flat, horizontal surface provides a convenient way to monitor root development in response to stimuli other than gravity, since the effect of gravity is not removed but is constant. Our setup provides a flat horizontal surface by overlaying the paper on a glass slide – which provides a flat surface – supported on a platform constructed from LEGO bricks – which ensures the surface is horizontal. The remarkable precision of LEGO bricks (molds have a tolerance of 5 µm or less [29]), together with their convenience, reusability, modularity, transparency, low cost, chemical inertness, and compatibility with autoclaving makes them nearly ideal building blocks for the rapid prototyping of structurally precise biological environments in the mm to cm scale [30]. Several setups can be arranged on a single flat leveled surface to ensure that all growth sheets in all setups are horizontal. Other options to control for gravitropism exist (e.g., using agravitropic mutants [31], growing plants in space [32] or in a clinostat [33]) but are considerably more demanding. ***Low cost***. There is a growing requirement to consider the cost of science from the beginning [34]. Our setups cost 8.78$ per plant, of which only 0.43$ is for disposable items. The setup does not require any equipment unless sterilization is required (in which case a class II biosafety cabinet is sufficient). ***High throughput***. High throughput plant experiments are typically conducted on gel in Petri dishes that (i) are capable of processing thousands of individual seeds/plants per week or month [35], [36], [37], (ii) can be setup in less than 1 minute, and (iii) can be stacked so that up to 5000 per m^2^ [35] can be fit in growth chambers. Our experimental units can be assembled in ∼1 to 2 minutes (see Movie S2 for a demonstration) and each of the parts can be prepared (i.e., autoclaved, cut, dissolved) in batches (we can assemble from scratch approximately 100 setups per person, per day). Each setup has a footprint of 60 cm^2^, or ∼167 units per m^2^. Although our setup does not have the throughput of Petri dishes, it provides control over humidity and is compatible with much larger plant sizes. ***Sterile***. Plant science research requires the growth and development of plants under sterile conditions [38]. All components are easily sterilized (the LEGO bricks, plastic cup, glass slide, MAGENTA box, gellan gum, salt and nutrient solutions are autoclaved, while the paper and plant seeds are soaked with 70% ethanol). After our setup is assembled in a sterile environment and sealed within the MAGENTA box, it can be transferred to a non-sterile environment without contamination. ***Suitable for any laboratory bench***. The advancement of life science in the 21^st^ century will require contributions from other disciplines [39] and developing world laboratories [40]. Facilitating these collaborations will require methods compatible with any laboratory bench in the world, regardless of discipline or resources. Our experiments were performed in handmade chambers (see Supporting Information S1) constructed from a wooden frame and aluminum foil. The purpose of the chambers was to provide uniform illumination of the plants, and prevent the establishment of thermal gradients. Plants were grown in our growth chambers underneath an array of 225 white LEDs so that the plants would receive ∼9000 lumens. ***Capable of supporting increasing levels of complexity***. The support of the seed is filter paper. This choice was influenced by the recent reports of ‘lab-on-paper’ technologies that have been developed to provide fluid manipulation [41], chemical reactions [42], and environments for microorganisms and cell cultures [43] in paper substrates. The combination of the platform presented here with the tools of paper microfluidics is beyond the scope of this communication, and will be the focus of future publications.

## Results and Discussion

The performance of the germination and growth environment was assessed by (i) its control over relative humidity, and (ii) its ability to yield high germination rates. Figure 2a shows the relative humidity (RH) measured 5 cm above the surface of the growth sheet (the approximate height of the cotyledons of a *B. rapa* plant after the hypocotyl straightens), as a function of the super saturated salt water solution held in the external container. All measurements were performed at 20°C: the measurements were made on a laboratory bench where the temperature was not stabilized (we estimate the error on the temperature to be ∼2°C). The RH can be controlled between 90.6 ± 0.9% (with KSO_4_, error is three standard errors, n = 3) and 56±8% (with LiCl, error is three standard errors, n = 3). The difference between these measured RH values and those expected from the respective saturated solutions – a super saturated LiCl solution in water should establish a RH of 12% – probably results from the fact that the atmosphere within the enclosed setup is exposed to both the saturated salt solution and the nutrient solution. Thereby, while the saturated salt solution is absorbing water from the atmosphere, reducing the RH, the nutrient solution is evaporating, increasing the RH. The steady state results in the observed RH. Of course, the above explanation implies that the observed RH will not only depend on the salt solution chosen to reduce RH, but also on the ratios between the areas of the exposed surfaces of the saturated salt solution and the nutrient solution in the setup. Broader ranges of RH should be accessible by changing the ratios of the exposed surfaces. The evaporation of the nutrient solution and the absorption of water by the saturated salt solution should increase the concentration of the nutrient solution over time. Our measurements indicate that the change is not detectable over the course of 15 days, at least when using NaCl as the saturated salt solution (see Supporting Information S1).

Figure 2b shows the germination rates for *B. rapa*, *T. aestivum*, and *Z. mays*, in our platform, compared to the germination rates reported by our seed source. The rates we obtained are remarkably close to the expected ones, especially considering that minimal effort was put into optimizing standard seed handling protocols for our platform (see Supporting Information S1 for details).

**Figure 3:**
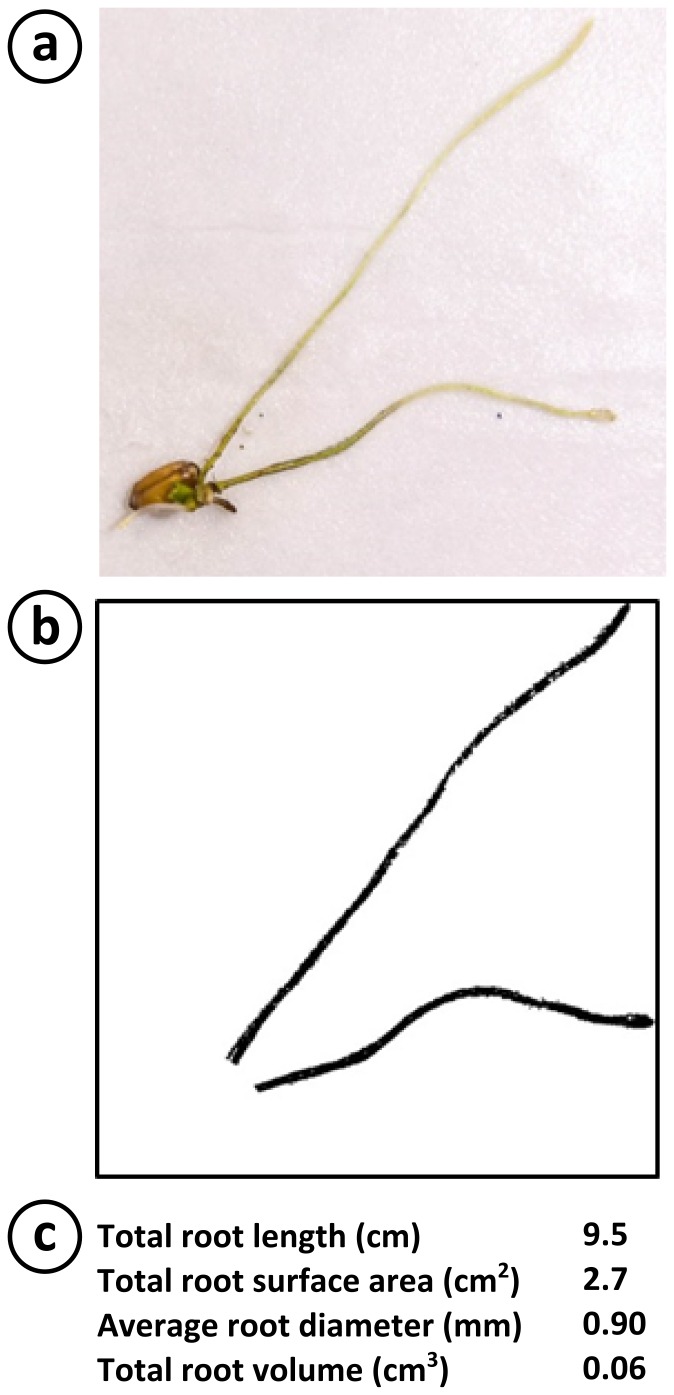
Example of root image analysis performed on roots grown on our experimental setup . **a**) Top-view photograph of a Wheat (*Triticum aestivum*) seedling grown for 7 days after the shoot has been removed. **b**) Modified version of the photograph in panel a) after the seed has been digitally removed, the color has been made black and white, the contrast has been maximised. **c**) Table of selected root parameters obtained by the analysis of the image in panel b) by WinRhizo.

The ability to visualize whole root systems will be increasingly important for understanding the responses of roots to stimuli, and breeding plants with desirable traits. Figure 3 demonstrates the use of our setup for the quantitative analysis of the whole root system of a *T. aestivum* seedling. The root system was photographed from above after the shoot is removed (Figure 3a). We increased the contrast of the image (details in Supporting Information S1) and removed the seed from consideration by superposing a white colored circle over it (Figure 3b). The resulting image was then analyzed with standard root-analysis software (in our case WinRhizo) yielding phenotypic data for the whole root system (Figure 3c).

## Conclusions

We addressed in this communication the challenge of providing a simple, inexpensive, and yet reproducible and capable apparatus for the observation of germination and seedling growth in sterile environments with controlled humidity.

The system we designed combines tools that are commonly used by plant scientists (e.g., filter paper, MAGENTA boxes) and others that are not (e.g., LEGO bricks) to fulfill a number of strict design requirements which include low cost, simplicity, structural precision, control over humidity, scalability to any plant size, and high throughput. Specifically, we demonstrated that the setup, as it is designed, (i) can grow plants for weeks, despite its planar geometry (the plants do not topple over but balance and anchor themselves with their roots), (ii) provides a constant supply of water, to the seed and root system, (iii) maintains a constant relative humidity between 91% and 56%, (iv) is capable of germination rates comparable to those expected from the species we tested (*B. rapa*, *T. aestivum*, and *Z. mays*), (v) enables sterile plant growth experiments in a non-sterile environment, (vi) facilitates imaging and image analysis of whole root systems, and (vii) cost 8.78$ (of which only 0.43$ are in non-reusable items) to buy and 1-2 minutes to assemble.

This platform represents one element of a series of integrated, simple, and reproducible tools that our group will be introducing to create highly controlled mm and cm-scale biological environments for plants and other organisms.

## Supporting Information

Supporting Information S1 **Figures S1-S16.** Figure S1. Construction of LEGO support. Figure S2. MAGENTA box containing LEGO support and glass slide. Figure S3. Preparation of nutrient solution and saturated sodium chloride solution. Figure S4. Preparation of cylinders and tweezers for sterilization. Figure S5. Form and dimensions of pump sheet (Whatman no. 1 filter paper). Figure S6. Form, dimensions and wax pattern of growth sheet (Whatman no. 1 filter paper). Figure S7. All consumables needed for the experiment. Figure S8. MAGENTA box containing 50 ml of super saturated sodium chloride. solution (liquid + powder NaCl). Figure S9. Mini cup (reservoir) box containing 25 ml of nutrient solution (x0.5 MS). Figure S10. Steps of MAGENTA box assembly with nutrient reservoir and pump sheet. Figure S11. Addition of the growth sheet paper. Figure S12. Gel and seed added to experimental setup, fully assembled setup. Figure S13. Growth chamber containing 18 setups. Figure S14. Photographs of a corn plant growing on a scaled-up version of the setup described in the manuscript. Larger sizes are possible. Figure S15. Measure of relative humidity (%), control without pump and growth paper and the experiment with pump and growth paper. Figure S16. The concentration of nitrates and ammonium ions measured in the pump sheet does not significantly change over time.
(PDF)

Movie S1 **Assembly of LEGO support for setup.**
(M4V)

Movie S2 Assembly of plant growth setup.
(M4V)
