# Supplementary material for: A Simple and Versatile 2-Dimensional Platform to Study Plant Germination and Growth under Controlled Humidity
Source: PLoS One. 2014 May 7;9(5):e96730. doi: 10.1371/journal.pone.0096730 (PMC4013031; doi:10.1371/journal.pone.0096730)
Supplement: Supporting Information S1 — Figures S1-S16. Figure S1. Construction of LEGO support. Figure S2. MAGENTA box containing LEGO support and glass slide. Figure S3. Preparation of nutrient solution and saturated sodium chloride solution. Figure S4. Preparation of cylinders and tweezers for sterilization. Figure S5. Form and dimensions of pump sheet (Whatman no. 1 filter paper). Figure S6. Form, dimensions and wax pattern of growth sheet (Whatman no. 1 filter paper). Figure S7. All consumables needed for the experiment. Figure S8. MAGENTA box containing 50 ml of super saturated sodium chloride. solution (liquid + powder NaCl). Figure S9. Mini cup (reservoir) box containing 25 ml of nutrient solution (x0.5 MS). Figure S10. Steps of MAGENTA box assembly with nutrient reservoir and pump sheet. Figure S11. Addition of the growth sheet paper. Figure S12. Gel and seed added to experimental setup, fully assembled setup. Figure S13. Growth chamber containing 18 setups. Figure S14. Photographs of a corn plant growing on a scaled-up version of the setup described in the manuscript. Larger sizes are possible. Figure S15. Measure of relative humidity (%), control without pump and growth paper and the experiment with pump and growth paper. Figure S16. The concentration of nitrates and ammonium ions measured in the pump sheet does not significantly change over time. (PDF) [file pone.0096730.s001.pdf]

A SIMPLE AND VERSATILE 2-DIMENSIONAL PLATFORM TO STUDY PLANT GERMINATION AND GROWTH UNDER CONTROLLED HUMIDITY

TOM SIZMUR[†‡§], KARA R. LIND[†], SAIDA BENOMAR[†‡], HANNAH VANEVERY[†⫠], LUDOVICO CADEMARTIRI[†‡⫠*]

† Department of Materials Science & Engineering, Iowa State University, Ames, Iowa, United States of America

‡ Ames Laboratory, US Department of Energy, Iowa State University, Ames, Iowa, United States of America

⫠ Department of Chemical & Biological Engineering, Iowa State University, Ames, Iowa, United States of America

§ current address: Department of Sustainable Soils and Grassland Systems, Rothamsted Research, Harpenden, Heartfordshire, United Kingdom

** Author to whom correspondence should be addressed: lcademar@iastate.edu*

SUPPORTING INFORMATION

MATERIALS

EQUIPMENT

Xerox Solid Ink Printer

Class II Biosafety Cabinet

Autoclave (Primus PSS5)

10 ml, 25 ml, 50 ml graduated cylinders

1 Liter glass bottles

50ml glass vials

Analytical balance

Stir plate, magnetic stir bar

Pipette 50-200 µl, pipette tips

Microwave

Oven capable of 80 °C

Inkscape

Digital Camera (Canon 50D, 100 Macro lens)

APPARATUS AND CONSUMABLES

20 x 20 cm sheet of Whatman no. 1 filter paper

50 x 50 mm glass slide (VWR CAT no. 48311-720)

Cocktail Mini Cubes, 2 inches tall, volume = 2 fl. Oz. (partyamerica.com SKU: 409974)

MAGENTA® vessel GA-7 77 mm × 77 mm × 97 mm (Sigma Aldrich SKU: V8505)

LEGO® bricks purchased from LEGO.com, Pick a Brick:

4 x Wall Element – TR 1X2X2 (Element ID: 4113028)

14 x Plate 1X2 (Element ID: 4167842)

Sodium chloride

Murashige & Skoog basal salt mixture with vitamins (Product code M519 from phytotechlab.com)

Seeds: Brassica rapa seeds (Wisconsin Fast Plants; Astroplants, *dwf1*), Lepidium sativum, Brassica oleracea (Broccoli), Solanum lycopersicum (tomato), Arabidopsis thaliana, Nicotina tabacum (Tobacco), Zea mays (Corn) and Triticum polonicum (Wheat).

Agar (Product code A111 from phytotechlab.com)

Tweezers

Petri dish

70% ethanol in spray bottle

Milli-Q deionized water

Sodium hypochlorite 5.25 / DI water (1:8 volumes)

Aluminum foil

Autoclave indicator tape

Nitrile gloves

Large Kimwipes

METHODS

SEED TREATMENT AND STERILIZATION

Before germination, seeds need to be treated and sterilized. Some seeds need also to be pre-chilled (or breaking dormancy, scarification) before germination.

For *Brassica rapa*, *Lepidium sativum*, *Brassica oleracea* (Broccoli), *Solanum lycopersicum* (tomato) and *Rosmarius officinalis* (rosemary):

Put the needed amount of seeds on a Petri dish.

Add 15 ml of Ethanol 70% (to cover all seeds).

Incubate for 15 min.

Wash three times with sterile DI water or sterile nutrient solution (MS).

For *Arabidopsis thaliana* and *Nicotina tabacum* (Tobacco):

Put the needed amount of seeds on a Petri dish.

Add like 15 ml of sodium hypochlorite 5.25% and DI water (1:8 volume).

Incubate for 15 min.

Wash three times with sterile DI water.

Pre-chill seeds at 4 °C for 24 hours in Gibberelic acid (also called Gibberellin A3 or GA3, 1 mg/ml on a new Petri dish between 3 layers of a sterile filter paper. Close the Petri dish using Parafilm.

For *Zea mays* (Corn) and *Triticum polonicum* (Wheat):

Put the needed amount of seeds on a Petri dish.

Add like 15 ml of sodium hypochlorite 5.25% and DI water (1:8 volume).

Incubate for 15 min.

Wash three times with sterile DI water.

Pre-chill seeds at 4 °C for 48 hours in Potassium nitrate 0.4 % (or sterile DI water) on a new Petri dish between 3 layers of a sterile filter paper. Close the Petri dish using Parafilm.

1. LEGO support (LEGO-based support)

Construction of LEGO-based support (see also the included video)

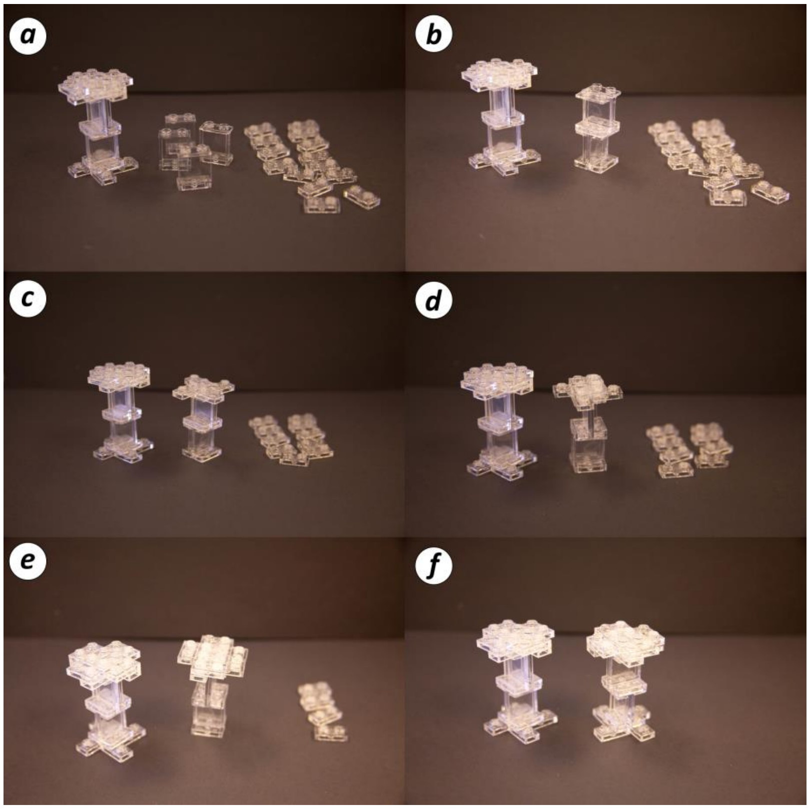

Figure S1. Construction of LEGO support.

Upon assembly of LEGO-based support, the tower is placed inside a MAGENTA box with the glass slide. A small piece of autoclave indicator tape is attached on the side of the MAGENTA box.

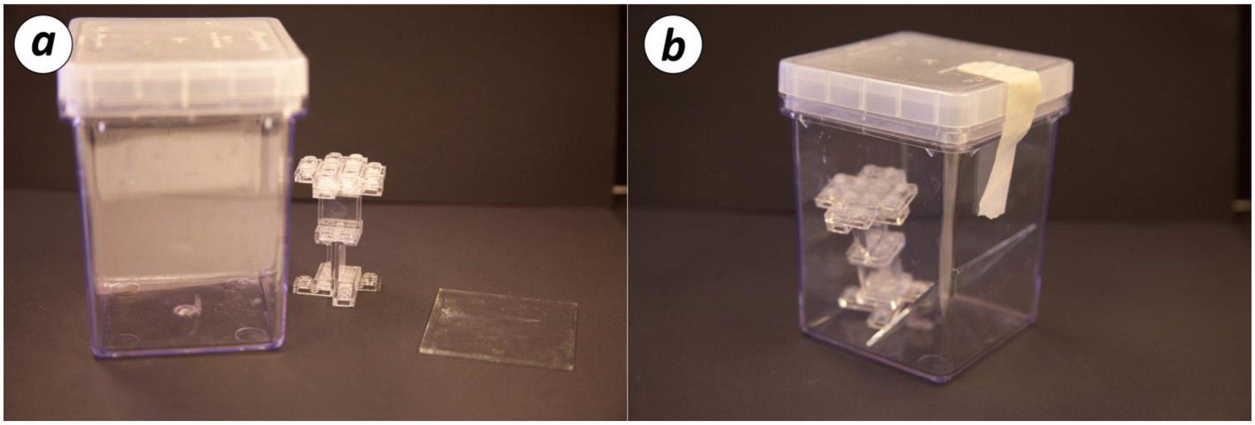

Figure S2. MAGENTA box containing LEGO support and glass slide.

MAGENTA boxes are autoclaved for 3.30 min at 132°C using a Primus PSS5 autoclave in cycle 2 for 15 min at 121°C.

2. Nutrient solution, Nutrient gel and saturated salt preparation

For the nutrient solution, an analytical balance is used to measure 2.215 g of Murashige & Skoog (MS) Basal Medium w/Vitamins. The MS is then added to 1 Liter of Milli-Q water in a glass bottle (2.215g/L=x0.5MS). Cap the bottle without fully tightening and place autoclave indicator tape across cap. Autoclave for 15 minutes at 121°C using cycle 6 of Primus PSS5 autoclave.

For the saturated salt solution, 500 g of NaCl (or another salt, depending on the desired relative humidity) is dissolved in 1 liter of Milli-Q water at room temperature. It works best to add NaCl over time using a stir plate and magnetic stir bar.

50mg of Agar and 22.15 mg MS is dissolved in 10 ml (0.5%) of Milli-Q water in a 50 ml glass vials (50mg/ml = 0.5%). The cap is closed without tightening and autoclave indicator tape is applied. The nutrient gel is then autoclaved similarly to salt and nutrient solutions.

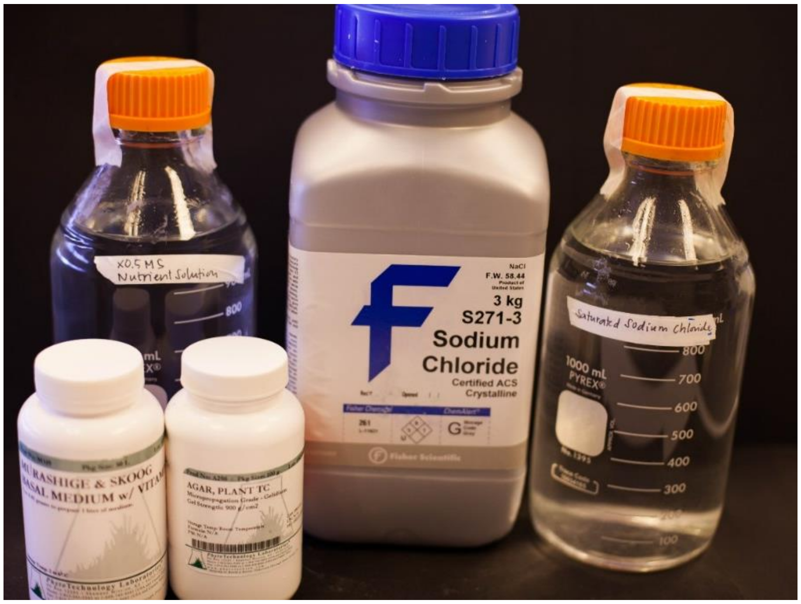

Figure S3. Preparation of nutrient solution and saturated sodium chloride solution.

50 ml, 25 ml, 10 ml measuring cylinders and tweezers are wrapped in aluminum foil and autoclave indicator tape then autoclaved on cycle 2 (same as MAGENTA boxes)

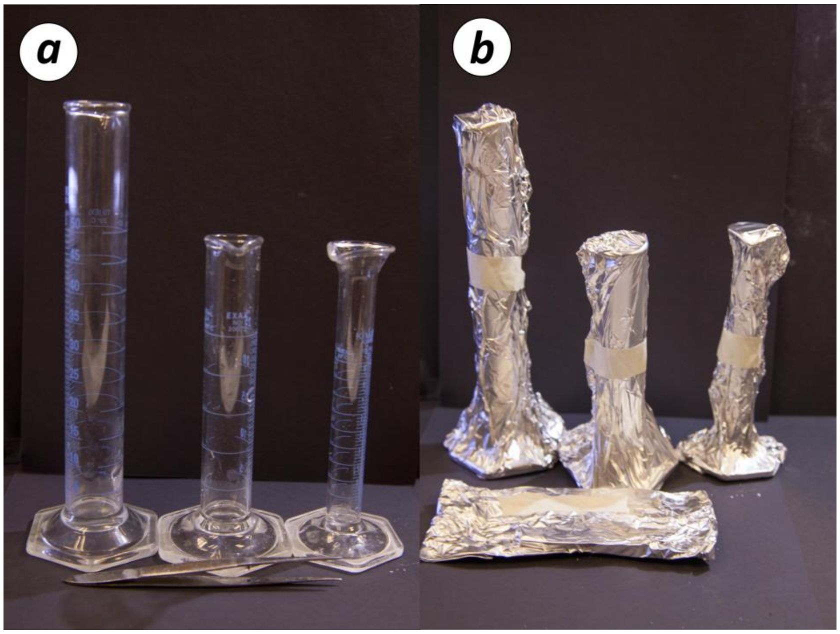

Figure S4. Preparation of cylinders and tweezers for sterilization.

3. Pump sheet

Using the Xerox printer, irregular hexagons are printed on Whatman no. 1 filter paper. Dimensions are displayed in figure S5. Each pump sheet should be cut on the inside of the printed lines.

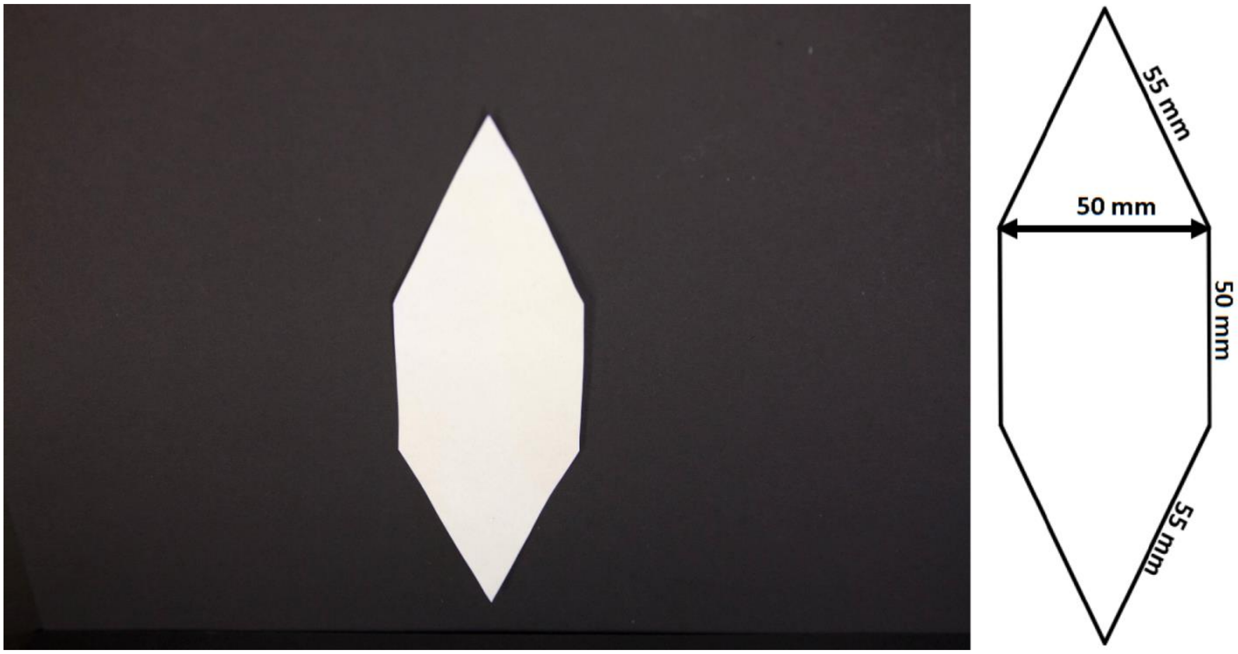

Figure S5. Form and dimensions of pump sheet (Whatman no. 1 filter paper).

4. Germination/Growth sheet

Using the Xerox printer, print a 50 X 50 mm square using a 1mm thick black border. The pattern used in this project is given below:

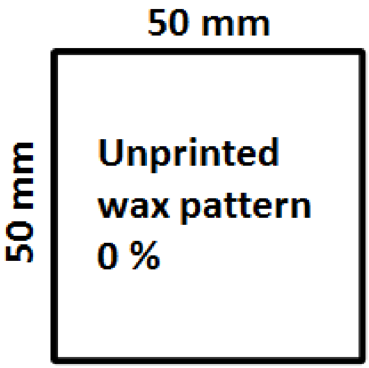

Figure S6. Form, dimensions and wax pattern of growth sheet (Whatman no. 1 filter paper).

5. Introducing material into the biosafety cabinet

Wearing nitrile gloves, the biosafety cabinet is sprayed with 70% ethanol. Wipes are then laid down after being soaked with 70% ethanol before being placed in the biosafety cabinet.
Each piece of growth and pump sheet is then sprayed on both sides with 70 % ethanol and placed on the wipes.

All items (the MAGENTA boxes, the wrapped measuring cylinders, the plastic bottles containing the sodium chloride) are sprayed with 70% ethanol before being placed in the biosafety cabinet.

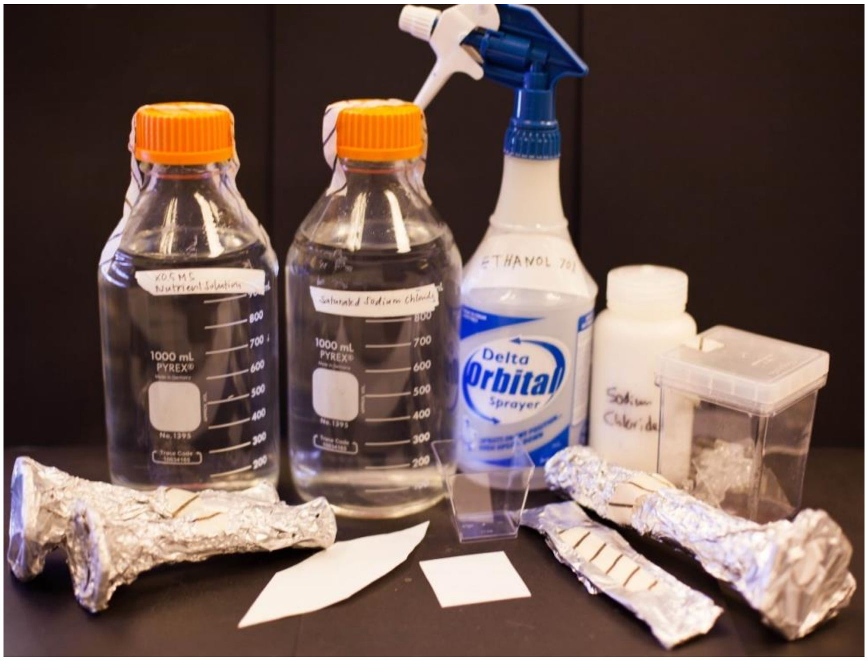

Figure S7. All consumables needed for the experiment.

The required number of seeds is placed on a Petri dish and 70% ethanol is added to cover the seeds before placing in the biosafety cabinet. When the ethanol has evaporated from the Petri dish, wash twice the seeds with 0.5xMS or sterile DI water and pour a small quantity of 0.5xMS salt solution on the Petri dish and keep the seeds moist throughout. When all the consumables are in the biosafety hood and ethanol has been given time to evaporate, assemble the experiment.

6. Assembling of the experimental apparatus (see also the video)

The MAGENTA box is opened and the glass slide and the LEGO-based support are removed and placed aside in the biosafety cabinet. NaCl is then poured into the MAGENTA box in excess. 50 ml of the saturated NaCl solution is poured into the MAGENTA box using the 50 ml graduated cylinder.

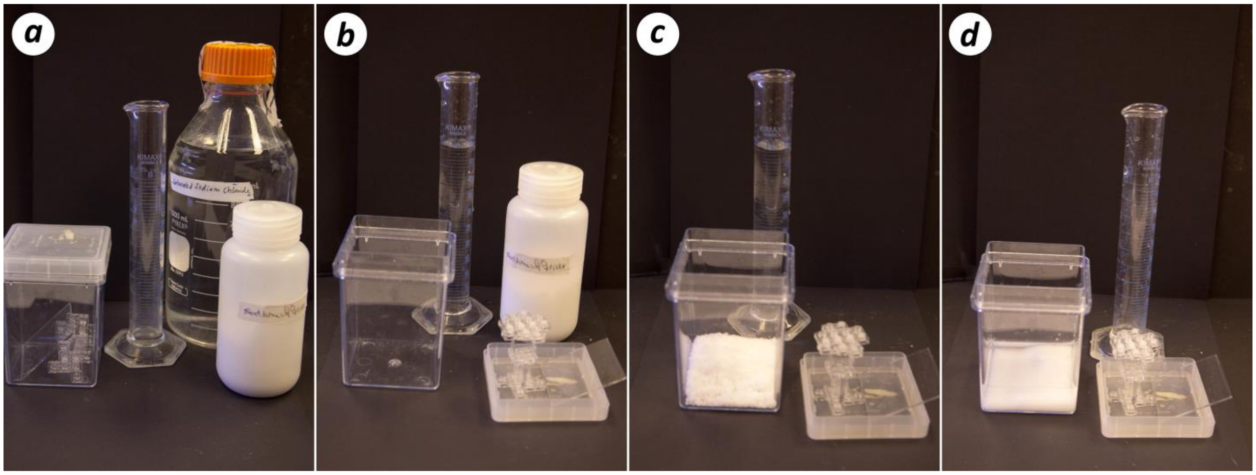

Figure S8. MAGENTA box containing 50 ml of super saturated sodium chloride solution (liquid + powder NaCl).

25 ml of the nutrient solution is poured into the nutrient vessel (cocktail mini cubes) using the 25 ml graduated cylinder. The LEGO-based support is then placed inside the nutrient vessel

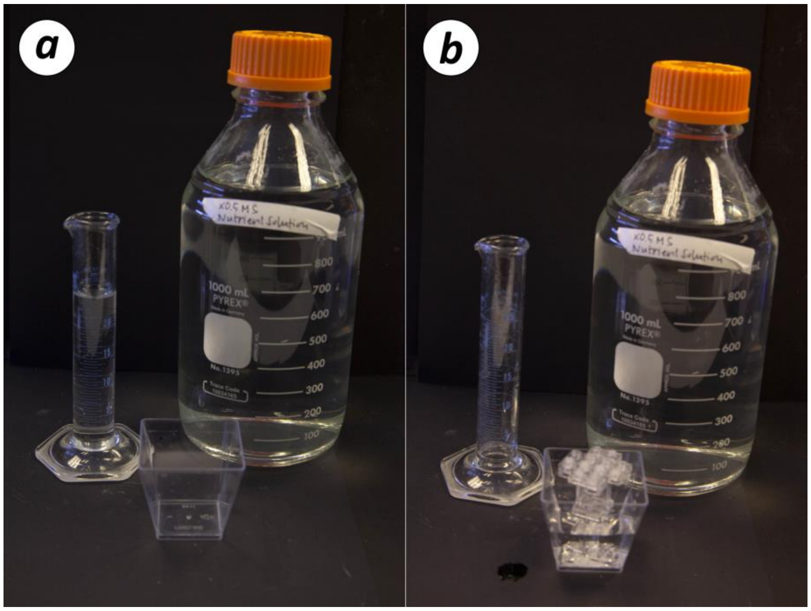

Figure S9. Mini cup (reservoir) box containing 25 ml of nutrient solution (x0.5 MS).

The nutrient vessel is then carefully lowered into the MAGENTA box containing the super saturated salt solution insuring no nutrient solution is lost and no super saturated salt solution enters the nutrient vessel.

The irregular hexagon is then folded around the glass slide so that the glass slide sits in the center and the two pointed ends point at an angle in the same direction.

The glass slide is lowered with the paper hexagon wrapped around it on to the LEGO-based support allowing for the tips of the hexagon to meet the nutrient solution. The position of the paper should be adjusted ensuring it covers the glass slide.

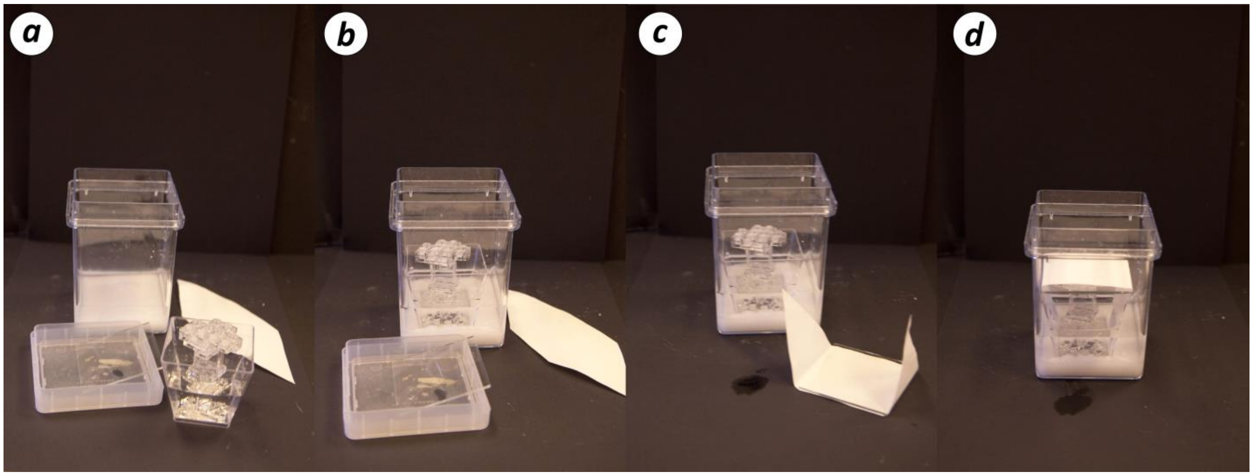

Figure S10. Steps of MAGENTA box assembly with nutrient reservoir and pump sheet.

To ensure the pump sheet is fully wet, 3ml of nutrient solution is added to the center using the 10 ml graduated cylinder then immediately the growth paper is placed on top. This step allows for the growth paper to become saturated upon placement and to be conformal to the underlying glass slide.

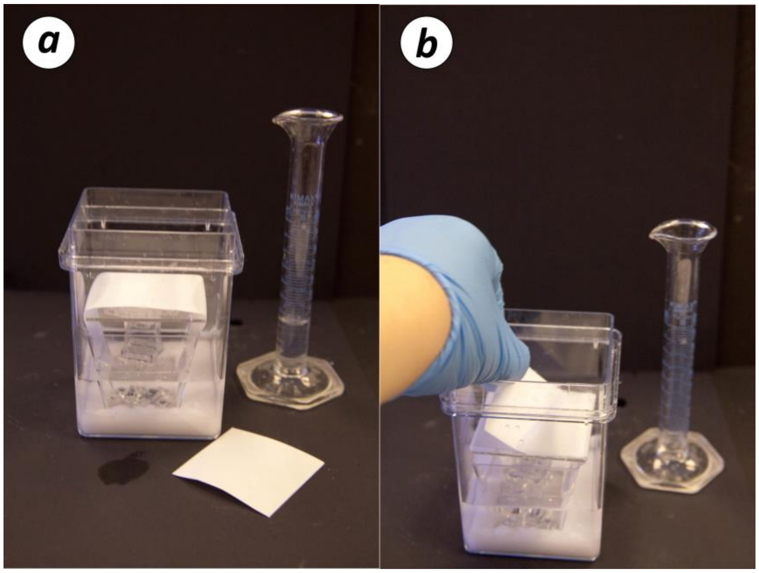

Figure S11. Addition of the growth sheet paper.

Using a pipette, pipette 50 µl of gel on the center of the paper (for *Brassica rapa* seed, larger amounts can be used for larger seeds: 200 µl for wheat and 500 µl for corn). If gel is not still liquid, it can be heated for 10 seconds using a microwave. The MAGENTA box lid is then placed back on the box before removing from the biosafety cabinet.

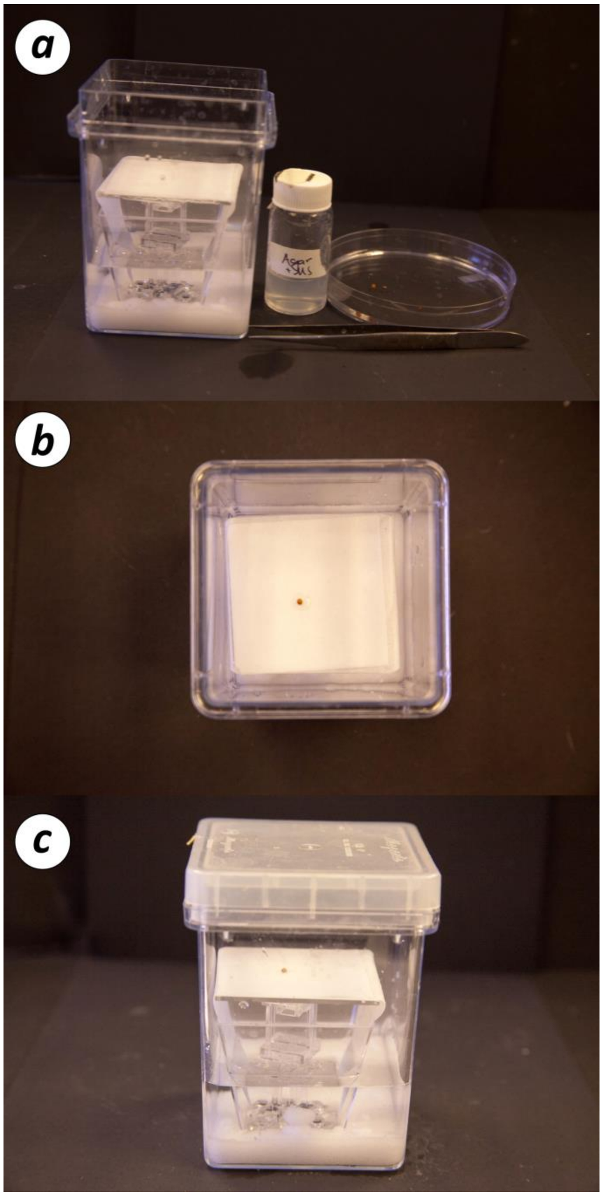

Figure S12. Gel and seed added to experimental setup, fully assembled setup

The box is then incubated for 1-3 weeks depending on the seed and experiment at room temperature under an array of 225 LED lights at a height of 20 cm above the MAGENTA box (so that the plants receive approximately 9000 lumens).

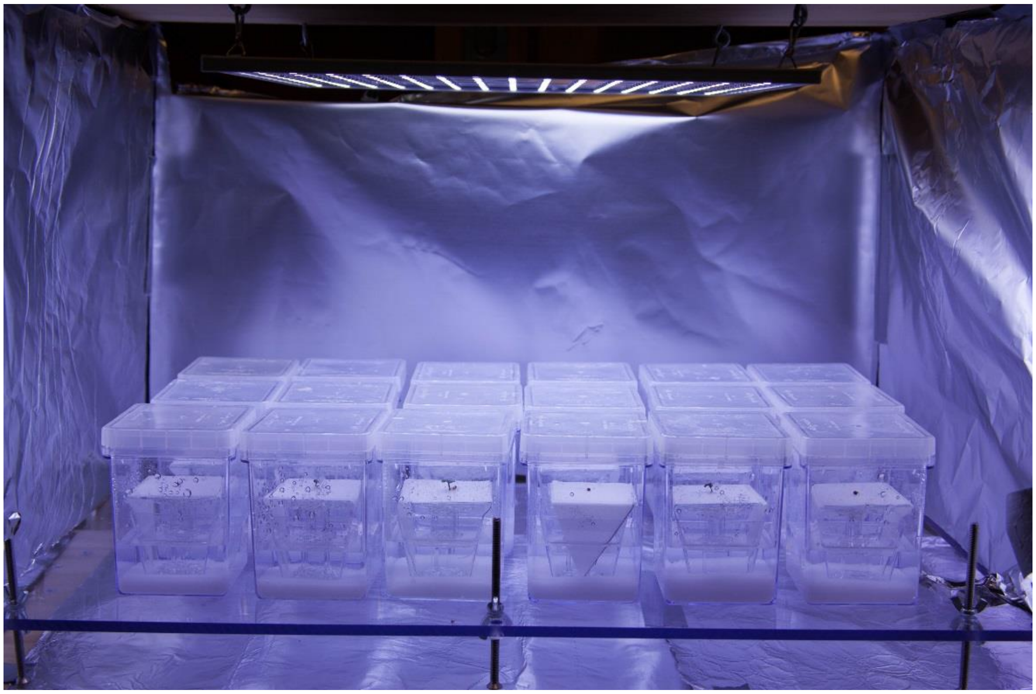

Figure S13: Growth chamber containing 18 setups.

7. Growth Chamber specifications

The growth chambers were custom built for the purpose of providing uniform illumination and protection from air drafts. They consist of a wooden framework, whose side walls are lined with aluminum foil to ensure homogeneous illumination (cf. Figure S13). The bottom of the chamber consists of a rigid plastic panel lifted from the ground by 4 screws at the corners. The screws can be regulated to level the plastic panel. The LED is hanging from the top of the chamber. The front panel is fitted with a hinge that allows for easy opening and closing of the growth environment.

8. Cost estimates (for setup displayed in Figure 1)

Item	Cost per batch	# of setups per batch	Cost per setup	Reusable	Disposable
Whatman no. 1 filter paper	$ 32.80	225	$ 0.15		
50 x 50 mm glass slide	$ 105.90	200	$ 0.53	$ 0.53	
Cocktail Mini Cubes	$ 3.49	10	$ 0.35	$ 0.35	
MAGENTA® vessel GA-7	$ 225.60	75	$ 3.01	$ 3.01	
LEGO® bricks: Wall Element – TR 1X2X2	$ 0.20	0.25	$ 0.80	$ 0.80	
LEGO® bricks: Plate 1X2	$ 0.10	0.071428571	$ 1.40	$ 1.40	
Sodium chloride	$ 45.32	20	$ 2.27	$ 2.27	
Murashige & Skoog basal salt mixture	$ 28.40	4000	$ 0.01		
Brassica rapa seeds	$ 12.50	50	$ 0.25		
Agar	$ 22.00	40000	$ 0.00		
Ethanol	$ 10.33	400	$ 0.03		
		Totals	$ 8.78	$ 8.35	$ 0.43

9. Scale Up

The setup described above can be easily scaled up to accommodate for larger plant sizes. Figure S14 shows an example of a 6.5 times increase in growing area obtained by using a combined LEGO-based support, a larger glass slide and larger sheets of filter paper.

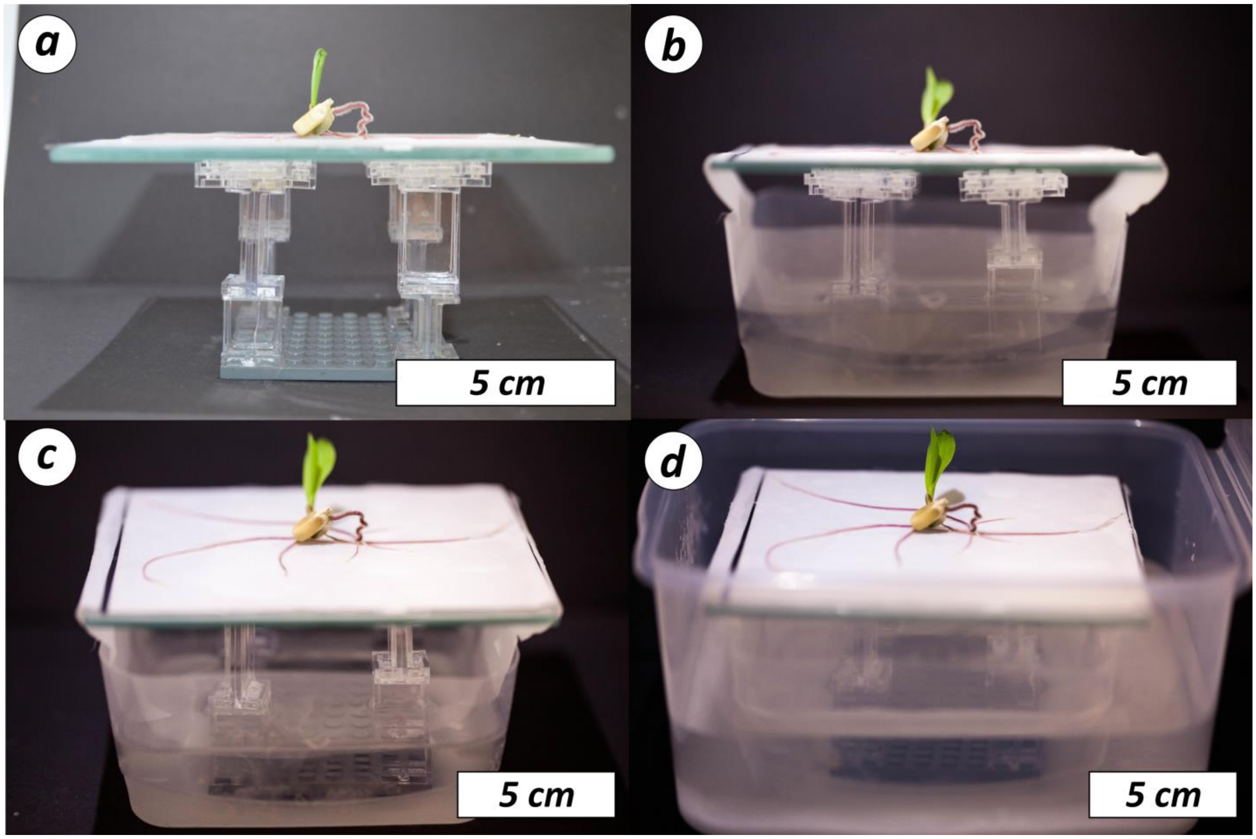

Figure S14. Photographs of a corn plant growing on a scaled-up version of the setup described in the manuscript. Larger sizes are possible.

RELATIVE HUMIDITY OF DIFFERENT SALTS

Prepare in a beaker (glassware) a super-saturated solution of salts by dissolving the amount of salt listed below in 100 ml of Milli-Q water. Pour the powder little by little with stirring at room temperature.
Potassium sulfate (50 g)
potassium nitrate (164.4 g)
potassium chloride (80 g)
sodium chloride (72 g)
potassium carbonate (224 g)
calcium chloride (300 g)
magnesium chloride (220 g)
lithium chloride (115 g).
Using the same protocol for plant germination see below:

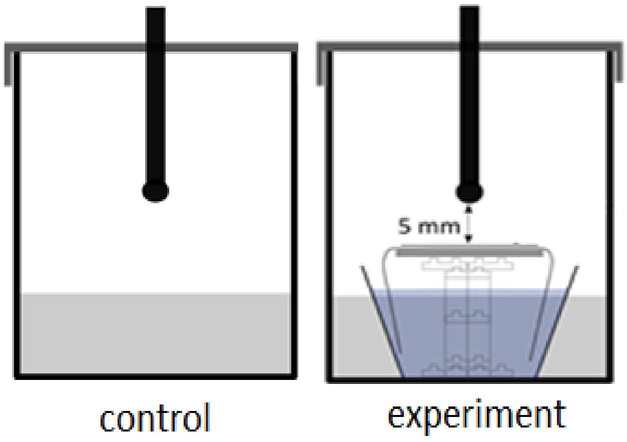

Figure S15. Measure of relative humidity (%), control without pump and growth paper and the experiment with pump and growth paper.

Cover the bottom of MAGENTA box by the one of the salt to test.
Add 50 ml of this salt
Use a MAGENTA box lid with hole
Introduce the hygrometer to measure the humidity (see figure S15 control).
Report the value indicated by the hygrometer when the temperature is stable.
Then, in the same box, put the cup (reservoir) with 25 ml nutrient solution and follow the same steps to put the growth paper.
Introduce the electrode to measure the humidity (see figure S15 experiment).
Report the value indicated by the relative humidity meter when the temperature is stable.

GERMINATION RATES

Seeds were sterilized and pretreated as described at the beginning of this document. 100 seeds for each species were germinated in the setups described in Figure 1. The numbers reported in Figure 2b were the highest germination rates we obtained for each species.

CONCENTRATION OF THE NUTRIENTS SOLUTION

The concentration of nitrates and ammonium ions were characterized in the nutrient solution after 0, 1, 6, 7, 8, 10 and 15 days, when using NaCl as the supersaturated salt. The results (n=3) are reported in Figure 15.

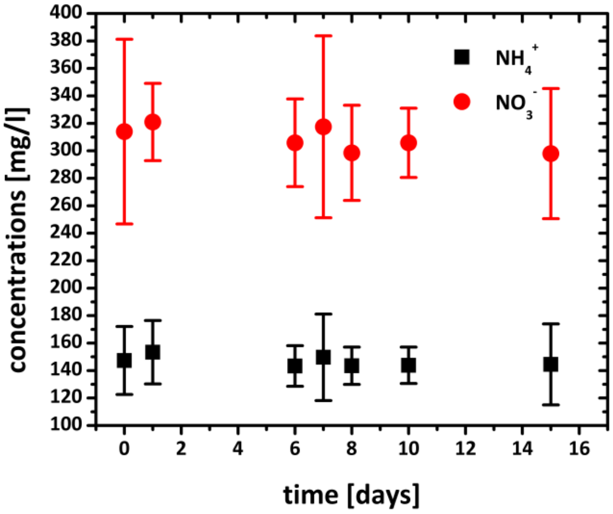

Figure S16. The concentration of nitrates and ammonium ions measured in the pump sheet does not significantly change over time

IMAGE ANALYSIS

The image of the corn seedling on the paper sheet (Figure 3a) was first converted to black and white, then processed by the "HDR-ish" filter of Google Picasa 3.0. The resulting image was subjected to a maximization of the Highlights and Shadows knobs, still in Picasa, resulting in a nearly binary image. The roots were then cut with a "Magic Wand" tool in Adobe Fireworks CS3 and pasted in a separate document with white background. The seed was covered and removed from further consideration by superposing a white circle over it. The resulting image was processed by WinRhizo yielding the root parameters described in Figure 3c.

LIMITATIONS AND FUTURE WORK

The platform described here is remarkably flexible, versatile, scalable, and, as future work from our group will demonstrate[1], capable of remarkable control over the physico-chemical parameters of the environment surrounding a plant and its root system. Nonetheless, it does present certain limitations, some of which are fundamental and some of which not so. *Temperature control*. The setup, in its current design stage, does not allow for the independent control of temperature. Each experiment can, however be freely moved from one controlled temperature environment to another without (i) physically disturbing the plant, (ii) affecting the atmospheric composition, (iii) compromising sterility, or (iv) affecting the orientation of the root with respect to gravity. *Germination at an angle*. While the root does anchor to the filter paper, the germination of species with large seeds cannot at present be accomplished at large angles (e.g., 90°) with the current design: the seed is not sufficiently adhering to the filter paper. This limitation could be easily overcome by overlying the seed with a second strip of paper, using the adhesion between this strip and the growth paper to hold the seed on the paper. *Inherently 2D geometry*. While the 2D geometry of the setup is remarkably convenient for the study of root development (e.g., localized application of stimuli, imaging and prototyping are substantially easier in a 2D configuration), 3D environments need to be developed as well to test any hypothesis originating from 2D experiments. Other work from our group [2] and others [3] is attempting to create 3D soil mimics for this purpose. *Incapable of generating very low humidity*. The current limit of the setup is ~55% RH, which is still high for any experiment that attempts to recreate very low humidity conditions. We believe this restriction is not a fundamental limit of the technique, and that RH values of ~10% are achievable with this same approach. *Instability of the nutrient concentration over time.* The current setup is vulnerable to relatively large changes in the concentration of the nutrients at the growth sheet due to the slow evaporation of the nutrient solution from it. Given the limited transport guaranteed by the pump sheet, the small volume of solution held in the pump sheet, and the relatively large distance between the top of the pump sheet and the nutrient reservoir, the concentration of nutrients can change over time. Modifications are being experimented with to provide not only a minimization of salt accumulation in the growth sheet but an effective constancy in the concentration of nutrients in contact with the root system. These modifications will require extensive characterization that is beyond the scope of this manuscript.

REFERENCES

1. Sizmur T, Lind KR, Benomar S, Vanevery H, Yuan B, et al. (2014): to be submitted.
2. Lind K, Sizmur T, Benomar S, Miller A, Cademartiri L (Submitted) LEGO® Bricks as Building Blocks for Centimeter and Millimeter Scale Biological Environments. Angewandte Chemie International Edition.

3. Downie H, Holden N, Otten W, Spiers AJ, Valentine TA, et al. (2012) Transparent Soil for Imaging the Rhizosphere. PLoS ONE 7: e44276.
